# Association Between Post-ERCP Pancreatitis and New-Onset Diabetes Mellitus: A Retrospective Cohort Study

**DOI:** 10.3390/jcm15103943

**Published:** 2026-05-20

**Authors:** Burak Furkan Demir, Ezgi Comoglu, Enes Seyda Şahiner, Zeki Mesut Yalin Kilic, Ihsan Ates

**Affiliations:** 1 Department of Internal Medicine, Ankara Bilkent City Hospital, Ankara 06800, Turkey; 2 Department of Internal Medicine, Etlik City Hospital, Ankara 06800, Turkey; 3 Department of Gastroenterology, Ankara Bilkent City Hospital, Ankara 06800, Turkey

**Keywords:** ERCP, Post-ERCP pancreatitis, new-onset diabetes mellitus, acute pancreatitis, risk factors

## Abstract

**Background/Objectives**: Acute pancreatitis is increasingly recognized as a risk factor for disturbances in glucose metabolism and the development of diabetes mellitus (DM). However, the long-term endocrine consequences of post-endoscopic retrograde cholangiopancreatography (ERCP) pancreatitis (PEP) remain poorly characterized. This study aimed to evaluate the association between post-ERCP pancreatitis and the risk of new-onset diabetes mellitus (NODM). **Methods**: This retrospective cohort study included patients who underwent ERCP between 2019 and 2024 at a tertiary referral center. New-onset diabetes mellitus was defined using laboratory data and International Classification of Diseases (ICD) diagnostic codes within one year after ERCP. Multivariable logistic regression adjusting for age, sex, hypertension, and coronary artery disease was performed. **Results**: A total of 2695 patients were included. Post-ERCP pancreatitis occurred in 165 patients (6.1%). New-onset diabetes developed in 9/165 patients (5.5%) in the PEP group and in 27/2530 patients (1.1%) in the non-PEP group. An increased incidence of new-onset diabetes was observed among patients who developed post-ERCP pancreatitis (crude OR 5.35, 95% CI 2.47–11.57; *p* < 0.001). In multivariable analysis adjusting for age, sex, hypertension, and coronary artery disease, post-ERCP pancreatitis remained significantly associated with new-onset diabetes in the fully adjusted model (adjusted OR 5.33, 95% CI 2.42–11.77; *p* < 0.001). The absolute risk increase was 4.39%, corresponding to a number needed to harm of 23. **Conclusions**: An increased incidence of new-onset diabetes was observed among patients who developed post-ERCP pancreatitis. This association remained significant after adjustment for baseline cardiovascular comorbidities. Although the absolute risk increase was modest, these findings may be clinically relevant. Because this was a retrospective study with a limited number of diabetes cases, the findings should be considered hypothesis-generating and require confirmation in prospective studies.

## 1. Introduction

Endoscopic retrograde cholangiopancreatography (ERCP) is widely used for the diagnosis and treatment of pancreaticobiliary disorders. Despite advances in technique and preventive strategies, post-ERCP pancreatitis (PEP) remains the most common complication of ERCP, with reported incidence rates ranging from 3% to 15% [1,2,3]. Although most cases are mild, PEP is associated with increased morbidity, prolonged hospitalization, and higher healthcare costs [1,3].

Diabetes mellitus secondary to diseases of the exocrine pancreas is classified as type 3c diabetes mellitus, also known as post-pancreatitis diabetes mellitus (PPDM), and may develop following acute pancreatitis, chronic pancreatitis, pancreatic surgery, or pancreatic malignancy [4,5]. In recent years, acute pancreatitis has increasingly been recognized as a risk factor for long-term disturbances in glucose metabolism. Previous studies and meta-analyses have reported that approximately 15% of patients develop diabetes within the first year after acute pancreatitis, with the cumulative incidence reaching 20–40% over longer follow-up periods [5,6,7,8].

The risk of diabetes appears to be higher in patients with severe pancreatitis, pancreatic necrosis, or recurrent attacks. However, several studies have shown that diabetes may also develop after mild acute pancreatitis, suggesting that even limited pancreatic inflammation may result in clinically relevant endocrine dysfunction [7,8,9,10].

Most previous studies have focused on acute pancreatitis of biliary, alcoholic, or idiopathic origin. In contrast, the long-term endocrine consequences of procedure-related pancreatic injury, such as post-ERCP pancreatitis, remain poorly characterized [3,11]. Because most cases of post-ERCP pancreatitis are clinically mild, they are often considered metabolically benign. However, this assumption has not been adequately evaluated in the literature [11,12].

Therefore, the aim of this study was to evaluate the association between post-ERCP pancreatitis and the risk of new-onset diabetes mellitus within one year following ERCP. To our knowledge, few studies have specifically evaluated new-onset diabetes following post-ERCP pancreatitis in a large ERCP cohort excluding patients with pre-existing diabetes and prior pancreatitis.

## 2. Methods

### 2.1. Study Design

This retrospective cohort study included consecutive patients who underwent ERCP between January 2019 and December 2024 at a tertiary referral center. The study was reported in accordance with the STROBE (Strengthening the Reporting of Observational Studies in Epidemiology) statement.

Patients with pre-existing diabetes mellitus, a history of acute or chronic pancreatitis, pancreatic malignancy, inflammatory bowel disease, newly diagnosed pancreaticobiliary malignancy after ERCP, or incomplete follow-up data were excluded. Patients without documented clinical follow-up within one year after ERCP were also excluded.

### 2.2. Definitions

#### 2.2.1. Post-ERCP Pancreatitis

Post-ERCP pancreatitis (PEP) was defined according to guideline criteria as new or worsened abdominal pain with serum amylase and/or lipase levels greater than three times the upper limit of normal within 24–48 h after ERCP, requiring hospitalization or prolongation of planned admission. The diagnosis was determined retrospectively from electronic medical records, discharge summaries, and laboratory results. The severity of pancreatitis was classified according to the revised Atlanta classification as mild, moderately severe, or severe based on the presence of organ failure and/or local complications [13].

#### 2.2.2. Pre-Existing Diabetes Mellitus

Pre-existing diabetes mellitus was identified using ICD diagnostic codes, antidiabetic medication use, and available laboratory data prior to ERCP. Baseline fasting glucose levels were within the normal range in included patients; however, pre-procedural HbA1c measurements were not available for all individuals.

#### 2.2.3. New-Onset Diabetes Mellitus

For the purposes of this study, new-onset diabetes mellitus was defined as either a documented diagnosis of diabetes based on ICD diagnostic codes within one year after ERCP or laboratory evidence of diabetes, defined as HbA1c ≥ 6.5% or fasting plasma glucose ≥ 126 mg/dL [14]. When available, laboratory data were used to support the diagnosis. Patients with insufficient diagnostic data were excluded to reduce misclassification.

### 2.3. Ethical Approval

The study was conducted in accordance with the principles of the Declaration of Helsinki and was approved by the Ethics Committee of the Ankara Bilkent City Hospital (approval no. TABED2/888/2025), and the requirement for informed consent was waived due to the retrospective design.

### 2.4. Statistical Analysis

Continuous variables were assessed for normality using visual methods (histograms and probability plots) and expressed as mean ± standard deviation or median (interquartile range), as appropriate. Normally distributed variables were compared using Student’s *t*-test, and non-normally distributed variables were compared using the Mann–Whitney U test. Categorical variables were compared using the chi-square test or Fisher’s exact test, as appropriate.

Risk estimates included relative risk (RR), odds ratio (OR), absolute risk difference (RD), and number needed to harm (NNH), all reported with 95% confidence intervals. Odds ratios were considered the primary measure of association.

Two multivariable logistic regression models were constructed using a forced-entry method to evaluate the association between post-ERCP pancreatitis and new-onset diabetes mellitus. Model 1 adjusted for age and sex. Model 2 additionally adjusted for hypertension and coronary artery disease, which were selected a priori as clinically relevant baseline comorbidities that could potentially confound the association between post-ERCP pancreatitis and new-onset diabetes. Given the limited number of outcome events, both models were intentionally kept parsimonious to avoid overfitting.

Model calibration was assessed using the Hosmer–Lemeshow goodness-of-fit test, and model explanatory power was evaluated using Nagelkerke R^2^. A two-tailed *p* value of <0.05 was considered statistically significant. Statistical analyses were performed using SPSS version 30 (IBM Corp., Armonk, NY, USA).

## 3. Results

### 3.1. Study Population

**Figure 1:**
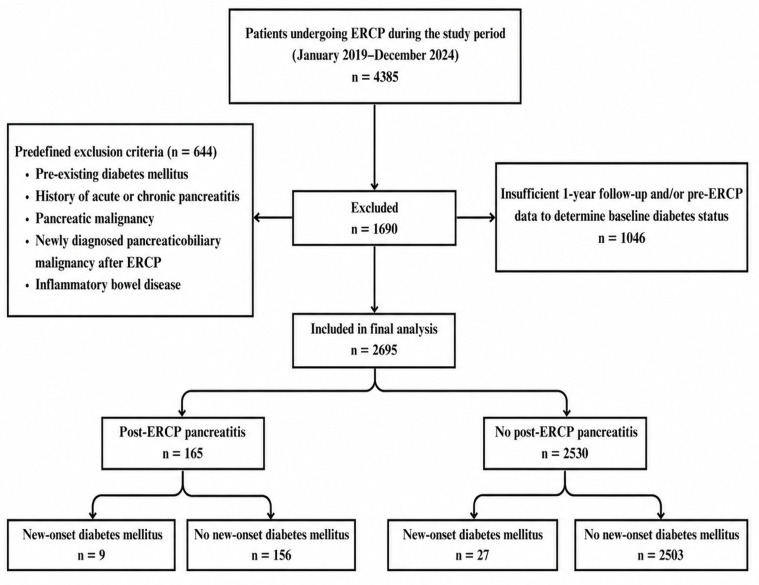
Patient selection flow diagram.

A total of 4385 patients who underwent ERCP during the study period were assessed for eligibility. Of these, 1046 patients were excluded because follow-up data were unavailable, and 644 patients were excluded for other predefined reasons. After application of the exclusion criteria, 2695 patients were included in the final analysis (Figure 1).

Post-ERCP pancreatitis developed in 165 patients (6.1%), while 2530 patients did not develop pancreatitis. New-onset diabetes mellitus developed in 9/165 (5.5%) patients in the PEP group and in 27/2530 (1.1%) patients in the non-PEP group.

Baseline characteristics, comorbidities, and procedural features of the study population are presented in Table 1. Age and sex distribution were similar between groups. However, hypertension (42.4% vs. 26.4%; *p* < 0.001), coronary artery disease (17.6% vs. 9.1%; *p* < 0.001), and chronic kidney disease (4.2% vs. 1.4%; *p* = 0.012) were more prevalent in the PEP group. Among procedural characteristics, endoscopic sphincterotomy (73.9% vs. 61.4%; *p* = 0.001), biliary stent placement (67.9% vs. 56.8%; *p* = 0.005), and pancreatic stent placement (16.4% vs. 7.1%; *p* < 0.001) were more frequent in the PEP group, whereas stone/sludge extraction was less frequent (64.8% vs. 73.0%; *p* = 0.023) (Table 1).

Continuous variables are presented as mean ± standard deviation and were compared using Student’s *t*-test. Categorical variables are presented as number (percentage) and were compared using the chi-square test or Fisher’s exact test, as appropriate. PEP = post-ERCP pancreatitis.

### 3.2. Risk of New-Onset Diabetes Mellitus

The risk of new-onset diabetes mellitus was higher among patients who developed post-ERCP pancreatitis. The relative risk was 5.11 (95% CI 2.44–10.69), and the unadjusted odds ratio was 5.35 (95% CI 2.47–11.57; *p* < 0.001). The absolute risk increase associated with post-ERCP pancreatitis was 4.39%, corresponding to a number needed to harm of 23 (Table 2).

Relative risk (RR), odds ratio (OR), absolute risk difference (RD), and number needed to harm (NNH) are presented. RR, OR, and RD are reported with 95% confidence intervals (CIs). RD is expressed as an absolute percentage difference. NNH was calculated as the reciprocal of the absolute risk difference and is presented as a point estimate.

### 3.3. Multivariable Analysis

In multivariable logistic regression analysis (Model 1), post-ERCP pancreatitis remained significantly associated with new-onset diabetes mellitus after adjustment for age and sex (adjusted OR 5.56, 95% CI 2.56–12.06; *p* < 0.001). In Model 2, which additionally adjusted for hypertension and coronary artery disease, post-ERCP pancreatitis remained significantly associated with new-onset diabetes (adjusted OR 5.33, 95% CI 2.42–11.77; *p* < 0.001). Age, sex, hypertension, and coronary artery disease were not independently associated with new-onset diabetes in the adjusted models (Table 3).

Model 1 was adjusted for age and sex. Model 2 was additionally adjusted for hypertension and coronary artery disease as clinically relevant baseline comorbidities. Both models were estimated using binary logistic regression with the forced-entry method. Model 1: Hosmer–Lemeshow *p* = 0.64, Nagelkerke R^2^ = 0.09. Dashes indicate variables not included in the respective model. OR = odds ratio; CI = confidence interval; HT = hypertension; CAD = coronary artery disease; PEP = post-ERCP pancreatitis.

### 3.4. Severity Analysis

Among patients who developed post-ERCP pancreatitis, most cases were mild (*n* = 153), while 12 patients had moderately severe pancreatitis. No cases of severe pancreatitis were observed. New-onset diabetes developed in 8/153 (5.2%) of patients with mild pancreatitis and 1/12 (8.3%) of those with moderately severe pancreatitis, with no statistically significant difference between severity groups (*p* = 0.53; Fisher’s exact test) (Table 4).

Categorical variables were compared using Fisher’s exact test due to the small number of events (*p* = 0.53). Values are presented as number and percentage. No cases of severe post-ERCP pancreatitis were observed. PEP = post-ERCP pancreatitis; DM = diabetes mellitus.

### 3.5. Exploratory Analysis

An exploratory analysis was performed among patients with post-ERCP pancreatitis to evaluate factors associated with new-onset diabetes. No statistically significant differences were observed between patients with and without diabetes in terms of demographic characteristics, laboratory values, comorbidities, or procedure-related factors. Due to the small number of diabetes cases (*n* = 9), these analyses were considered exploratory and hypothesis-generating (Table 5).

Continuous variables are presented as median (interquartile range) and were compared using the Mann–Whitney U test. Categorical variables are presented as number (percentage) and were compared using Fisher’s exact test. Due to the small number of diabetes cases (*n* = 9), these analyses were considered exploratory and hypothesis-generating.

## 4. Discussion

This study showed that patients who developed post-ERCP pancreatitis had a higher incidence of new-onset diabetes mellitus within one year compared with patients who underwent ERCP without pancreatitis. The incidence of diabetes was approximately five times higher in the PEP group, and this association remained statistically significant after adjustment for age, sex, hypertension, and coronary artery disease. The consistency between the unadjusted odds ratio, the age- and sex-adjusted estimate, and the fully adjusted model supports the robustness of the observed association. However, residual confounding cannot be fully excluded.

Post-ERCP pancreatitis remains the most frequent adverse event of ERCP. Its pathogenesis is multifactorial and includes papillary trauma, repeated cannulation or pancreatic duct instrumentation, hydrostatic or chemical injury from contrast injection, and thermal injury during sphincterotomy, all of which may trigger local pancreatic inflammation [1,3,15]. Although many episodes are mild, PEP remains clinically important because it may prolong hospitalization, increase morbidity, and increase healthcare utilization [1,3]. In the literature, the incidence of PEP in unselected ERCP cohorts has generally been reported in the range of 3–15%, although estimates vary according to study design, patient mix, and the proportion of high-risk procedures [16,17,18,19]. In selected elderly or clinically complex populations, somewhat higher rates have also been reported [20,21]. Overall, the 6.1% PEP incidence observed in our cohort is consistent with the published literature.

One of the most notable findings of this study is that an increased incidence of diabetes was also observed among patients with mild post-ERCP pancreatitis. Although diabetes incidence was numerically higher in moderately severe pancreatitis than in mild pancreatitis, the difference was not statistically significant. Because most PEP cases in our cohort were mild, most diabetes cases occurred in patients with mild pancreatitis; however, this should not be interpreted as evidence that mild pancreatitis carries a higher absolute risk than more severe pancreatitis, as the study was not powered to detect differences across severity groups.

Previous studies have shown that diabetes may develop in approximately 15–20% of patients within the first year after acute pancreatitis and in up to 20–40% during longer follow-up periods [6,7,8,9]. The risk appears to be higher in patients with severe pancreatitis, pancreatic necrosis, or recurrent attacks [7,9]. Compared with the higher diabetes rates reported after severe or recurrent acute pancreatitis, the 5.5% one-year NODM rate observed after PEP in our cohort appears lower, although still clinically meaningful given that most PEP cases were mild. A preliminary report has also suggested a possible association between post-ERCP pancreatitis and new-onset diabetes, although data remain limited [11]. Several studies have also shown that diabetes may occur after mild pancreatitis, supporting the concept that even limited pancreatic inflammation may impair endocrine function [7,9,10]. Our findings are consistent with these observations and suggest that procedure-related pancreatitis may also have clinically relevant long-term endocrine consequences.

The mechanisms linking pancreatitis and diabetes are likely multifactorial and may include inflammatory injury to pancreatic beta cells, systemic inflammatory responses leading to insulin resistance, microvascular ischemia, and alterations in the incretin axis [5,22]. Pancreatic exocrine dysfunction has also been associated with the development of diabetes after pancreatitis, suggesting an interaction between exocrine and endocrine pancreatic insufficiency [12,23]. In the setting of ERCP, mechanical trauma to the pancreatic duct, hydrostatic injury from contrast injection, and chemical irritation may trigger pancreatic inflammation affecting both exocrine and endocrine pancreatic tissue [1,3]. Even when the clinical course is mild, inflammatory injury to islet cells may contribute to impaired glucose metabolism over time.

From a clinical perspective, although the relative risk increase observed in this study was substantial, the absolute risk increase was modest (4.39%), corresponding to a number needed to harm of 23. Given the large number of ERCP procedures performed worldwide, even a modest absolute risk increase may still be clinically relevant. These findings suggest that patients who develop post-ERCP pancreatitis may represent a higher-risk group for dysglycemia and may benefit from closer metabolic follow-up during the first year after ERCP [6,8].

This study has several limitations. First, the retrospective design may have introduced selection bias and unmeasured confounding. Second, the number of new-onset diabetes cases was relatively small (*n* = 36 total). This limited the number of variables that could be included in the multivariable analysis, increased the risk of model overfitting, and reduced the statistical power of subgroup and severity-based analyses. Third, detailed metabolic risk factors such as body mass index and family history of diabetes were not consistently available due to the retrospective design. In addition, although baseline fasting glucose values were within the normal range in included patients, pre-procedural HbA1c measurements were not available for all individuals. Fourth, although patients without documented follow-up were excluded, patients who developed post-ERCP pancreatitis may still have undergone closer clinical and laboratory monitoring than those without pancreatitis. This may have increased the likelihood of detecting new-onset diabetes and introduced detection bias. Future prospective studies using standardized post-ERCP metabolic follow-up protocols may help reduce this potential source of bias. Fifth, no cases of severe post-ERCP pancreatitis were observed in this cohort, limiting the evaluation of diabetes risk across the full severity spectrum. Finally, this was a single-center study, which may limit the generalizability of the findings.

While the association between acute pancreatitis and diabetes has been well established, data specifically evaluating diabetes risk after post-ERCP pancreatitis remain limited. To our knowledge, this is one of the few studies specifically evaluating this risk, and among the few to show that the association persists after adjustment for selected baseline comorbidities. However, given the retrospective design and limited number of outcome events, these findings should be considered hypothesis-generating and should be confirmed in prospective multicenter studies with more comprehensive metabolic risk assessment.

## 5. Conclusions

Post-ERCP pancreatitis, even when clinically mild, was associated with a higher incidence of new-onset diabetes within one year in this study. This association remained statistically significant after adjustment for selected baseline comorbidities, including hypertension and coronary artery disease. Although a causal relationship cannot be established in this retrospective cohort, these findings suggest that patients who develop post-ERCP pancreatitis may represent a higher-risk group for dysglycemia, for whom metabolic monitoring during follow-up may be considered. In practical terms, approximately one additional case of diabetes may occur for every 23 patients who develop post-ERCP pancreatitis. Further prospective studies with longer follow-up are needed to better define the long-term endocrine consequences of post-ERCP pancreatitis.

## Figures and Tables

**Table 1:** Baseline characteristics, comorbidities, and procedural features according to post-ERCP pancreatitis status.

*p* Value	No PEP (*n* = 2530)	PEP (*n* = 165)	
			Demographics
0.899	59.5 ± 18.0	59.3 ± 17.8	Age, mean ± SD
0.208	1194 (47.2%)	69 (41.8%)	Male sex, *n* (%)
<0.001	27 (1.1%)	9 (5.5%)	New-onset diabetes, *n* (%)
			Comorbidities
<0.001	669 (26.4%)	70 (42.4%)	Hypertension, *n* (%)
<0.001	231 (9.1%)	29 (17.6%)	Coronary artery disease, *n* (%)
0.589	58 (2.3%)	5 (3.0%)	Cerebrovascular disease, *n* (%)
0.012	35 (1.4%)	7 (4.2%)	Chronic kidney disease, *n* (%)
0.119	81 (3.2%)	9 (5.5%)	Hyperlipidemia, *n* (%)
0.206	65 (2.6%)	7 (4.2%)	Hypothyroidism, *n* (%)
0.022	40 (1.6%)	7 (4.2%)	Dementia, *n* (%)
			Procedure characteristics
0.001	1553 (61.4%)	122 (73.9%)	Endoscopic sphincterotomy, *n* (%)
0.023	1847 (73.0%)	107 (64.8%)	Stone/sludge extraction, *n* (%)
0.005	1436 (56.8%)	112 (67.9%)	Biliary stent placement, *n* (%)
<0.001	179 (7.1%)	27 (16.4%)	Pancreatic stent placement, *n* (%)
0.778	214 (8.5%)	15 (9.1%)	Balloon dilation, *n* (%)
0.947	79 (3.1%)	5 (3.0%)	Biopsy, *n* (%)

**Table 2:** Risk of new-onset diabetes mellitus associated with post-ERCP pancreatitis.

95% CI	Estimate	Measure
2.44–10.69	5.11	Relative Risk
2.47–11.57	5.35	Odds Ratio
0.9–7.9%	4.39%	Absolute Risk Increase
—	23	Number Needed to Harm

**Table 3:** Multivariable logistic regression analysis for predictors of new-onset diabetes mellitus.

Model 2 (Age + Sex + HT + CAD)			Model 1 (Age + Sex)			Variable
*p*	95% CI	OR	*p*	95% CI	OR	
Primary predictor						
<0.001	2.42–11.77	5.33	<0.001	2.56–12.06	5.56	Post-ERCP pancreatitis
Covariates						
0.716	0.97–1.02	1	0.829	0.98–1.02	1	Age (per year)
0.104	0.89–3.52	1.77	0.066	0.96–3.73	1.89	Male sex
0.556	0.32–1.85	0.77	—	—	—	Hypertension
0.125	0.81–5.58	2.13	—	—	—	Coronary artery disease

**Table 4:** New-onset diabetes mellitus according to post-ERCP pancreatitis severity.

DM Rate (%)	New-Onset DM, *n*	*n*	PEP Severity
5.20%	8	153	Mild
8.30%	1	12	Moderately severe

**Table 5:** Exploratory analysis of factors associated with new-onset diabetes among patients with post-ERCP pancreatitis.

*p* Value	No DM (*n* = 156)	DM (*n* = 9)	Variable
Demographics and laboratory			
0.83	62 (45–74)	64 (53–66)	Age, median (IQR)
0.32	96 (78–126)	120 (99–124)	LDL, median (IQR)
0.20	121 (87–155)	141 (133–146)	Triglycerides, median (IQR)
0.07	16 (10–35)	8 (7–14)	CRP, median (IQR)
0.98	8.8 (7.2–11.0)	8.7 (7.6–10.4)	WBC, median (IQR)
0.41	1075 (676–1742)	789 (678–1023)	Amylase, median (IQR)
0.43	1700 (984–2950)	1206 (988–1566)	Lipase, median (IQR)
0.41	7 (6–10)	8 (7–11)	Hospital stay, median (IQR)
Comorbidities and procedure			
0.49	64 (41.0%)	5 (55.6%)	Male sex, *n* (%)
1.00	66 (42.3%)	4 (44.4%)	Hypertension, *n* (%)
0.66	27 (17.3%)	2 (22.2%)	Coronary artery disease, *n* (%)
1.00	7 (4.5%)	0	Chronic kidney disease, *n* (%)
1.00	9 (5.8%)	0	Hyperlipidemia, *n* (%)
1.00	26 (16.7%)	1 (11.1%)	Pancreatic stent placement, *n* (%)
0.72	105 (67.3%)	7 (77.8%)	Biliary stent placement, *n* (%)
0.16	99 (63.5%)	8 (88.9%)	Stone/sludge extraction, *n* (%)
0.70	116 (74.4%)	6 (66.7%)	Endoscopic sphincterotomy, *n* (%)
1.00	5 (3.2%)	0	Biopsy, *n* (%)

## Data Availability

Data from the study are not openly available to other researchers due to protected patient information. Further inquiries can be directed to the corresponding author.
